# Arsenic Exposure and Neurodevelopmental Disorders in Children: A Cross‑Sectional Study

**DOI:** 10.5334/aogh.4874

**Published:** 2025-12-29

**Authors:** Claudia López, Paola Rubilar, María P Muñoz, Macarena Hirmas-Adauy, Verónica Iglesias

**Affiliations:** 1 Programa de Magíster en Salud Pública, Escuela de Salud Pública, Facultad de Medicina, Universidad de Chile, Santiago, Chile; 2 Centro de Epidemiología y Políticas de Salud, Facultad de Medicina Clínica Alemana, Universidad del Desarrollo, Santiago, Chile; 3 Programa de Doctorado en Salud Pública, Escuela de Salud Pública, Facultad de Medicina, Universidad de Chile, Santiago, Chile; 4 Escuela de Salud Pública, Facultad de Medicina, Universidad de Chile, Santiago, Chile; 5 Centro para la Prevención y el Control del Cáncer (CECAN), Santiago, Chile

**Keywords:** arsenic, neurodevelopmental disorders, children, exposure

## Abstract

*Introduction:* Arsenic exposure has been identified as a possible risk factor for neurodevelopmental disorders (NDDs). In Arica, research has been conducted to relate arsenic exposure to the prevalence of attention deficit hyperactivity disorder (ADHD). However, highlighting the need to explore other events, such as autism spectrum disorder (ASD), this study aimed to evaluate the association between current urinary arsenic concentration and the prevalence of NDDs in children from Arica.

*Methods:* A cross‑sectional study was conducted using secondary data from the FONIS project #SA22I0119. The sample consists of 450 children born between 2013 and 2016. The outcome variable, diagnosis of NDDs, was measured through parent self‑reporting. The exposure variable corresponds to the current concentration of urinary inorganic arsenic, corrected by creatinine. A logistic regression model adjusted for confounding variables was used.

*Results:* According to parent self‑report, the prevalence of ADHD was 9.1%, ASD 5.3%, and NDDs 12%. The mean urinary inorganic arsenic concentration was 19.8 μg/g creatinine, and 7.6% of the children had levels ≥35 μg/g creatinine. After adjusting for tutors’ education, number of household members, sex, and indigenous origin, those children with urinary arsenic ≥ 35 μg/g creatinine were more likely to present some NDDs (OR: 2.93; 95% CI 1.11, 7.75). For ADHD, the association was also elevated (OR = 3.85; 95% CI 1.44, 10.29).

*Conclusion:* The findings suggest an association between arsenic exposure and the prevalence of NDDs in children. These results contribute to the evidence of arsenic’s effect on the neurodevelopment of the child population.

## Introduction

Arsenic has been ranked as the most significant potential threat to human health, measured by its abundance, toxicity, and potential for human exposure [1]. At the brain level, damage is associated with weight reduction, decreased glial and neuronal cells, and alterations in neurotransmitter systems [2]. It crosses the hematoplacental barrier, accumulating in fetal epithelial tissue [3]. The neurotoxic effect has been attributed to its ability to induce oxidative stress [4, 5], causing DNA damage [1, 6] and altering the dopaminergic system [5]. Likewise, the hippocampal NMDA receptors, which play a role in synaptic plasticity, learning, and memory, are affected, which can lead to neurobehavioral disorders and cognitive dysfunctions [1, 6]. In the child population, it has been associated with cognitive problems, academic performance [7], attention problems, impulsivity [5, 8], and neurodevelopmental disorders (NDDs) [9].

NDDs reflect atypical brain development, emerging in early life or during the school years [10], and potentially leading to impairment in various spheres of children’s lives [11, 12]. Up to 10% of the child population is estimated to have one or more NDDs [12], such as attention deficit hyperactivity disorder (ADHD) and autism spectrum disorder (ASD) [9]. ADHD is the most diagnosed neurobehavioral disorder in school‑aged children [13]. Globally, the prevalence is estimated at 7.6% in children between 3 and 12 years old (95% CI 6.1, 9.4) [14], while in Chile, the prevalence in children and adolescents has been reported to reach 10% [15]. In the case of ASD, globally, it is estimated that between 1% and 2% present it at different levels [10]; however, between 2002 and 2008, a 7.8% increase was revealed [16]. Nationally, in 2021, the prevalence of ASD in the urban population was estimated at 1.95% (95% CI 0.81, 4.63) [17].

ADHD is characterized by a persistent pattern of inattention or hyperactivity and impulsiveness [18], and ASD corresponds to permanent deficits in communication and social interaction, as well as restricted, repetitive, and inflexible patterns of behavior and interests [18]. Both conditions have strong genetic correlates [9], and it has been proposed that the two disorders share some neurochemical and neurodevelopmental pathways [12]. Internationally, these two conditions have co‑occurrence prevalences ranging from 30% to 70% [12], and both ADHD and ASD have multifactorial etiopathogenesis resulting from a very complex interaction between genetic, biological, chemical, psychological, environmental, and social factors [10, 19].

It is estimated worldwide that over 200 million children under five are not reaching their developmental potential due to exposure to multiple risk factors, including poverty, malnutrition, unsafe domestic environments, and early exposure to environmental contaminants [20]. In Arica, in a previous study, the prevalence of ADHD self‑reported by parents was 6.4% [5]. In a study conducted at La Greda School in Ventanas, a geographical area permanently exposed to contaminants such as arsenic, lead, manganese, zinc, and copper [21], the prevalence of ADHD or learning disorder (LD) was 23% [5, 15].

The presence of arsenic in Arica has different origins. In water, it results from the natural dissolution of minerals present in the rocky bed through which water flows before its use, as well as high concentrations in groundwater [22]. In line with the World Health Organization (WHO) guidelines, national regulations have established a maximum limit of 10 μg/L of arsenic in drinking water [23], a standard currently met in urban areas such as Arica. Regarding soil, during the 1980s, 20,000 tons of sludge containing hazardous waste with high levels of lead, arsenic, cadmium, and mercury were stored in an area where, years later, social housing was built, exposing around 15,000 residents [24]. This toxic material was later relocated, and Law 20.590 [25] (the Polymetal Law) was enacted, establishing a comprehensive health program for populations exposed to polymetals. As part of the committed actions, the Environmental Health Center was established in 2009 to provide medical care to those affected by heavy metal contamination [25].

Although internationally, some studies have reported a relationship between arsenic exposure and the development of ASD and ADHD, evidence remains limited. In a previous study conducted in Arica, only data from a group of children enrolled at the Environmental Health Center of Arica between 2009 and 2015 were available [5]. The current research takes an additional step by considering children from the entire city, including variables not previously measured, and incorporating ASD into NDDs to add evidence to support the findings. Thus, the objective of this study focused on evaluating the association between current urinary arsenic concentration and the prevalence of NDDs (ASD or ADHD) in children from the city of Arica.

## Methods

### Design, population, and sample

The cross‑sectional analytical study was based on data from the FONIS project #SA22I0119 titled “Exposure to arsenic and its association with proinflammatory cytokines in children born between 2013 and 2016 in the city of Arica.” The study population corresponds to 1,644 mothers who participated in the study “Prevalence of arsenic in pregnant women and lead in newborns at Dr. Juan Noe Hospital,” conducted by the Arica and Parinacota Health Authority between 2013 and 2016. The Health Authority provided this database through a collaboration agreement with the Faculty of Medicine of the University of Chile. As part of the FONIS project, contact with these mothers was reinitiated at the end of 2022 to invite them to participate in the follow‑up. The sample size of the study framework corresponds to 450 children [26].

### Variables

The outcome variable was the diagnosis of NDDs, specifically ASD and ADHD. These conditions were identified based on parental self‑reports collected through a questionnaire. For this study, a new binary variable was created, classifying participants as having an NDD if at least one of the two self‑reported conditions (ASD or ADHD) was self‑reported.

The exposure variable was the current concentration of urinary inorganic arsenic measured in the main study [26]. Briefly, a trained nursing assistant collected the spot urine sample and stored it until it was sent to a certified private laboratory. The inductively coupled plasma mass spectrometry method was used to determine the concentration of each arsenic species: arsenite (AsIII), arsenate (AsV), monomethylarsonic acid (MMA), dimethylarsinic acid (DMA), and arsenobetaine. The detection limit for each analyte was 0.1 µg/L. The sum of the inorganic arsenic species (AsIII and AsV) and the methylated arsenic species (MMA and DMA) is referred to herein as “In‑As.” The urinary In‑As concentrations were corrected by creatinine concentration to account for urine dilution.

Covariates were obtained from the questionnaire applied to the parents. Sociodemographic variables included age, sex, belonging to indigenous people, educational level of the mother or tutor, and the number of household members. Child education‑related variables considered were history of grade repetition, belonging to the School Integration Program (SIP), attendance at a special or differential school, and type of educational establishment. Environmental exposure variables included screen time, household pesticide use, drinking and cooking water sources, pavement in the yard or street in front of the house, exposure to secondhand smoke in the last 72 h, and whether the mother or father is a beneficiary of Law 20,590.

### Data collection

REDCap software was used to collect data in the framework study. The questionnaire application and sample collection were conducted by two work teams, each comprising a nursing assistant and a nurse. Children whose mothers or tutors agreed to participate in the study during the pre‑enrollment and enrollment phases were subsequently visited at home between June and August 2023. During these visits, informed consent was obtained, a spot urine sample was collected, and the questionnaire was administered.

### Statistical analysis

Quantitative variables were summarized using medians and interquartile ranges for descriptive analysis, while categorical variables were described in terms of frequencies. The Wilcoxon rank‑sum test (Mann–Whitney test) and the Kruskal–Wallis test were used to compare arsenic concentrations according to participants’ characteristics. Pearson’s chi‑square test and Fisher’s exact test (applied when expected frequencies were below five observations) were used to compare the prevalence of ADHD and ASD according to sociodemographic and exposure characteristics. Both unadjusted and adjusted logistic regression models were performed to evaluate the association between urinary In‑As concentration and the prevalence of ASD, ADHD, and NDDs. The results of these analyses are presented in tables that describe the odds ratios (OR) along with their respective 95% confidence intervals (95% CI). Confounding variables were identified by performing a directed acyclic graph (DAG) and by assessing the percentage of change in the effect estimate. The Akaike information criterion (AIC) was used to evaluate the goodness of fit. A lower AIC value indicates that the model best fits the data with the fewest possible parameters. An interaction term (arsenic* belonging to indigenous people) was included in the model, and its statistical significance was evaluated. Data were analyzed with the STATA v.18.0 statistical software.

### Ethical aspects

The relevant ethics committees authorized both the study framework and the current study. The study framework was reviewed and approved by the Scientific Ethics Committee of the Facultad de Medicina de la Universidad del Desarrollo (#81‑2022). Participants who agreed to participate in the research were informed and guided through the consent and assent process. The current study was approved by the Human Research Ethics Committee of the Facultad de Medicina, Universidad de Chile (#177‑2023).

## Results

The studied sample consisted of 450 children. We had complete data for sociodemographic characteristics, urinary In‑As concentration, and NDDs. The mean age was 7.8 years (SD 0.68), and 49.6% were males. Regarding belonging to indigenous people, 38% were identified as Aymara descendants and 7.3% as other indigenous groups. In terms of child education‑related characteristics, 31% had repeated a grade, 25.3% required special educational needs (SEN) support as they currently participate in the SIP, and 29.6% had attended a special or differentiated school at some point. According to parent reports, the prevalence of ASD and ADHD was 5.3% and 9.1%, respectively, and the diagnosis of any NDDs was 12.0% (Table 1).

**Table 1:** Sociodemographic and exposure characteristics of the study sample.

SOCIODEMOGRAPHIC CHARACTERISTICS	*N* (%)
Child sex	
Female	227 (50.4)
Male	223 (49.6)
Age (years)	
7–8	379 (84.2)
9–10	71 (15.8)
Belonging to indigenous peoples	
No	246 (54.7)
Aymara	171(38.0)
Other	33 (7.3)
Mother’s or tutor’s education	
0–8 years	30 (6.7)
9–12 years	217 (48.2)
≥13 years	203 (45.1)
People living in the same house	
2–4	210 (46.7)
5–14	240 (53.3)
History of grade repetition	
Yes	14 (3.1)
Attending the School Integration Program	
Yes	110 (25.3)
Attendance at special or differential school	
Yes	133 (29.6)
Screen exposure (day hours)	
0–2	121 (26.9)
3–10	329 (73.1)
Household pesticide use	
Yes	214 (47.6)
Father or mother beneficiary of the Polymetal Law	
Yes	72 (16.0)
ASD reported by parents	
Yes	24 (5.3)
ADHD reported by parents	
Yes	41 (9.1)
NDD (ASD or ADHD) reported by parents	
Yes	54 (12.0)

*Note*: ASD, autism spectrum disorder; ADHD, attention deficit hyperactivity disorder; NDD, neurodevelopmental disorders.

A two‑ to threefold higher prevalence of ASD, ADHD, and NDDs was observed in males compared to females. Similarly, a higher prevalence was observed in children with special educational needs who belong to SIPs (eight‑ to tenfold higher than those who do not attend). Children who have attended a special or differential school at some point also report a higher prevalence of NDDs (Table 2).

**Table 2:** Prevalence of ASD, ADHD, and NDD according to sociodemographic and exposure characteristics.

VARIABLES	ASD *N* = 24		ADHD *N* = 41		NDD (ASD OR ADHD) *N* = 54	
	*N*	(%)	*N*	(%)	*N*	(%)
Child sex						
Male	18	(8.1)	28	(12.6)	39	(17.5)
Female	6	(2.6)	13	(5.7)	15	(6.6)
*p*‑value*^a^*	0.010		0.012		<0.001	
Belonging to indigenous peoples						
Aymara	6	(3.5)	11	(6.4)	15	(8.8)
Other	4	(12.1)	3	(9.1)	5	(1.2)
No	14	(5.7)	27	(11.0)	34	(13.8)
*p*‑value*^a^*	0.099		0.274		0.250	
Mother’s or tutor’s education						
0–8 years	1	(3.3)	1	(3.3)	1	(3.3)
9–12 years	15	(6.9)	23	(10.6)	32	(14.8)
≥13 years	8	(3.9)	17	(8.4)	21	(10.3)
*p*‑value*^a^*	0.377		0.442		0.131	
Attending the School Integration Program						
Yes	19	(17.3)	32	(29.1)	40	(36.7)
No	5	(1.5)	9	(2.8)	14	(4.3)
*p*‑value*^a^*	<0.001		<0.001		<0.001	
Attendance at special school						
Yes	14	(10.5)	22	(16.5)	30	(22.6)
No	10	(3.2)	19	(6.0)	24	(7.6)
*p*‑value*^a^*	0.001		<0.001		<0.001	
Screen exposure (day hours)						
0–2	7	(5.8)	12	(9,9)	15	(12.4)
3–10	17	(5.2)	29	(8.8)	39	(11.9)
*p*‑value*^a^*	0.796		0.719		0.875	
Frequency of household pesticide use						
Doesn’t use	12	(5.1)	27	(11.4)	31	(13.1)
At least once a year	4	(6.0)	2	(3.0)	6	(9.0)
Monthly	4	(4.9)	8	(9.8)	10	(12.2)
Weekly	2	(3.9)	4	(7.7)	5	(9.6)
Daily	2	(15.4)	0	(0.0)	2	(15.4)
*p*‑value*^a^*	0.512		0.217		0.861	
Father or mother beneficiary of Polymetal Law						
Yes	5	(5.5)	7	(9.7)	10	(13.9)
No	19	(6.9)	33	(9.5)	43	(12.4)
Doesn’t know	0	(0.0)	1	(3.3)	1	(3.3)
*p*‑value*^a^*	0.431		0.658		0.286	

*Note*: ASD, autism spectrum disorder; ADHD, attention deficit hyperactivity disorder; NDD, neurodevelopmental disorders.

*^a^* Pearson’s chi‑square test and Fisher’s exact test were used for categorical variables.

The median and interquartile range (IQR) of the urinary In‑As concentration were 17 and 12–23.9 μg/g creatinine. This measure does not follow a normal distribution and was dichotomized into ≥35 μg/g creatinine, corresponding to the reference value established in the clinical guidelines of the Polymetal Law [27]. Notably, 7.6% of children have values ≥35 μg/g. When observing the arsenic level in different groups, it was slightly higher in those aged 7 to 8 years, those belonging to the Aymara indigenous people, those with a mother or tutor with primary schooling, those using pesticides at home, those using well or river water for drinking and cooking, and those without pavement in the yard or front street of the house (Table 3).

**Table 3:** Description of urinary inorganic arsenic level corrected for creatinine according to sociodemographic and exposure variables.

VARIABLES	URINARY IN‑AS (μG/G)		
	*N*	MEDIAN (P_25th_–P_75th_)	*P*‑VALUE*^a^*
Child sex	450		0.834
Female	227	16.9 (12.0–24.2)	
Male	223	17.0 (11.8–23.5)	
Age (years)	450		0.041
7–8	379	17.7 (12.2–24.3)	
9–10	71	15.0 (11.5–21.4)	
Belonging to indigenous peoples	450		0.022
No	246	15.7 (11.2–22.4)	
Aymara	171	18.7 (13.5–25.2)	
Other	33	17.3 (12.7–24.7)	
Mother’s or tutor’s education	450		0.002
0–8 years	30	21.6 (12.1–33.2)	
9–12 years	217	17.8 (12.7–26.0)	
≥13 years	203	15.3 (11.2–21.7)	
Number of household members	450		0.375
2–4	210	16.5 (11.7–22.9)	
5–14	240	17.4 (12.1–24.6)	
2–14*^b^*		Rho = 0.027	0.562
History of grade repetition	450		0.612
No	436	17.0 (12.0–23.9)	
Yes	14	16.4 (14.0–31.9)	
Attending the School Integration Program	450		0.302
No/Doesn’t know	340	16.7 (12.0–23.4)	
Yes	110	18.1 (12.1–26.0)	
Attendance at special school	450		0.270
No	317	16.3 (12.0–23.5)	
Yes	133	18.2 (12.1–24.3)	
Screen exposure (day hours)	450		0.399
0–2	121	17.8 (13.3–22.8)	
3–10	329	16.7 (12.0–23.9)	
0–10*^b^*		Rho = −0.081	0.086
Frequency of household pesticide use	450		0.033
Doesn’t use	236	15.4 (11.7–21.6)	
At least once a year	67	19.5 (13.7–25.9)	
Monthly	82	19.4 (11.1–25.0)	
Weekly	52	17.9 (13.0–24.3)	
Daily	13	19.3 (13.3–27.2)	
Source of drinking water	450		0.007
Bottled water	382	16.7 (11.7–22.9)	
Public water network or water tanker	66	18.9 (13.7–27.4)	
Well water	2	60.5 (28.2–92.8)	
Source of water for cooking	450		0.008
Bottled water	44	15.5 (10.2–20.9)	
Public water network or water tanker	399	16.9 (12.1–24.2)	
Well water	7	27.4 (20.4–60.7)	
Paved floor in the house’s patio or front garden	450		0.001
No	72	20.4 (13.9–32.0)	
Yes	378	16.5 (11.7–22.7)	
Paved road in front of the house	449		0.061
No	77	19.1 (13.3–28.2)	
Yes	372	16.8 (11.9–23.1)	
Exposure to SHS in the last 72 h	450		0.929
No	370	17.4 (12.0–23.9)	
Yes	80	15.9 (12.8–23.8)	
Father or mother beneficiary of Polymetal Law	450		0.455
No/Doesn’t know	378	17.0 (12.0–23.3)	
Yes	72	17.1 (12.3–24.8)	
ASD reported by parents	450		0.205
No	426	16.9 (12.0–23.5)	
Yes	24	21.6 (12.6–29.2)	
ADHD reported by parents	450		0.659
No	409	16.9 (12.1–23.5)	
Yes	41	17.3 (11.8–27.3)	
NDD (ASD or ADHD) reported by parents	450		0.423
No	396	16.9 (12.0–23.2)	
Yes	54	18.6 (12.1–26.8)	

*Note*: P_25th_–P_75th_ percentile; ASD, autism spectrum disorder; ADHD, attention deficit hyperactivity disorder; NDD, neurodevelopmental disorders; SHS, secondhand smoke.

*^a^* Wilcoxon (Mann–Whitney) test for dichotomous variables and Kruskal–Wallis test for categorical variables.

*^b^* Spearman correlation between continuous variables (presented categorized in the table).

In the unadjusted logistic regression analysis (Table 4), significant associations were observed between NDDs and male sex (OR = 3.00; 95% CI 1.60, 5.61), children who regularly attend SIP (OR = 13.31; 95% CI 6.87, 25.77); children who have participated at a special or differential school at some point (OR = 3.56; 95% CI 1.99, 6.36); and children who attend a public school (OR = 1.95; 95% CI 1.10, 3.45). These same variables were also associated with ASD and ADHD. Regarding exposure variables, no significant association was observed except when analyzing the group with current In‑As levels ≥ 35 μg/g creatinine, which had a threefold increased likelihood of presenting ADHD (OR = 2.91; 95% CI 1.18, 7.18) and a twofold increase in the likelihood of presenting any NDDs (OR = 2.04; 95% CI 0.84, 4.93).

**Table 4:** Bivariate analysis between the prevalence of ASD, ADHD, and NDD according to sociodemographic and exposure variables.

SOCIODEMOGRAPHIC VARIABLES	ASD	ADHD	NDD
	OR (95% CI)	OR (95% CI)	OR (95% CI)
Child sex			
Female	Reference	Reference	Reference
Male	3.23 (1.26, 8.31)	2.36 (1.19, 4.69)	3.00 (1.60, 5.61)
Belonging to indigenous peoples			
No	Reference	Reference	Reference
Aymara	0.60 (0.23, 1.60)	0.56 (0.27, 1.16)	0.60 (0.32, 1.14)
Other	2.29 (0.70, 7.41)	0.81 (0.23, 2.84)	1.11 (0.40, 3.08)
Mother’s or tutor’s education			
≥13 years	Reference	Reference	Reference
9–12 years	1.81 (0.75, 4.37)	1.30 (0.67, 2.51)	1.50 (0.83, 2.70)
0–8 years	0.84 (0.10, 6.97)	0.38 (0.05, 2.94)	0.30 (0.04, 2.31)
History of grade repetition			
No	Reference	Reference	Reference
Yes	1	2.86 (0.76, 10.69)	2.06 (0.56, 7.63)
Attending the School Integration Program			
No/Doesn´t know	Reference	Reference	Reference
Yes	13.99 (5.08, 38.49)	15.09 (6.92, 32.90)	13.31 (6.87, 25.77)
Attendance at special school			
No	Reference	Reference	Reference
Yes	3.61 (1.56, 8.35)	3.11 (1.62, 5.96)	3.56 (1.99, 6.36)
Type of school			
Private or semi‑private school	Reference	Reference	Reference
Public	1.71 (0.75, 3.91)	2.06 (1.08, 3.94)	1.95 (1.10, 3.45)
Screen exposure (day hours)			
0–2	Reference	Reference	Reference
3–10	0.89 (0.36, 2.20)	0.88 (0.43, 1.78)	0.95 (0.50, 1.79)
Frequency of household pesticide use			
At least once a year	Reference	Reference	Reference
Monthly	0.81 (0.19, 3.36)	3.51 (0.72, 17.14)	1.41 (0.49, 4.11)
Weekly	0.63 (0.11, 3.58)	2.71 (0.48, 15.40)	1.08 (0.31, 3.76)
Daily	2.86 (0.47, 17.57)	1	1.85 (0.33, 10.37)
Source of drinking water			
Bottled water	Reference	Reference	Reference
Public water network or water tanker	1.57 (0.56, 4.35)	1.21 (0.51, 2.87)	1.18 (0.55, 2.55)
Well water	1	1	1
Source of water for cooking			
Bottled water	Reference	Reference	Reference
Public water network or water tanker	1.23 (0.28, 5.40)	1.44 (0.43, 4.87)	1.43 (0.49, 4.18)
Well water	1	1	1
Paved floor in the house’s patio or front garden			
No	Reference	Reference	Reference
Yes	1.35 (0.39, 4.66)	0.92 (0.39, 2.16)	1.11 (0.50, 2.46)
Paved road in front of the house			
No	Reference	Reference	Reference
Yes	0.78 (0.28,– 2.14)	1.23 (0.50, 3.03)	1.04 (0.49, 2.23)
Exposure to SHS in the last 72 h			
No	Reference	Reference	Reference
Yes	1.59 (0.61, 4.13)	1.34 (0.61, 2.93)	1.38 (0.69, 2.75)
Father or mother beneficiary of Polymetal Law			
No/Doesn’t know	Reference	Reference	Reference
Yes	1.41 (0.51, 3.91)	1.09 (0.46, 2.56)	1.22 (0.58, 2.56)
Current urinary In‑As concentration corrected by creatinine μg/g			
In‑As < 35 μg/g	Reference	Reference	Reference
In‑As ≥ 35 μg/g	1.12 (0.25, 4.97)	2.91 (1.18, 7.18)	2.04 (0.84, 4.93)

*Note*: Simple logistic regression was used; ASD, autism spectrum disorder; ADHD, attention deficit hyperactivity disorder; NDD, neurodevelopmental disorders; SHS, secondhand smoke; OR, odds ratio; CI, confidence interval.

As previously mentioned, confounding variables were initially identified through a DAG, which showed that socioeconomic status needed to be accounted for as a confounder. However, this variable was not directly measured in the study framework, so proxy variables such as the mother’s or tutor’s education and the number of household members were used (model 2, Table 5). Then, confounding variables were verified using the percentage change in the effect estimate, and the mother’s or tutor’s education, the number of household members, belonging to indigenous peoples, and sex were identified (model 3, Table 5). Finally, the AIC was used to determine that model 3 provided the best fit to the data. In the adjusted analysis, children with urinary In‑As levels ≥ 35 μg/g creatinine had 2.93 times the likelihood of developing any NDDs (95% CI 1.11–7.75). They also have 3.85 times the likelihood of developing ADHD (95% CI 1.44–10.29). No association with ASD was observed (Table 5). In this sample, belonging to indigenous people did not modify the effect of arsenic on NDDs (data not shown).

**Table 5:** Association between arsenic exposure and ASD, ADHD, and NDD according to parents’ reports.

	MODEL 1*^b^*		MODEL 2*^c^*		MODEL 3*^d^*	
ARSENIC*^a^*	OR (95% CI)	*P*‑VALUE	OR (95% CI)	*P*‑VALUE	OR (95% CI)	*P*‑VALUE
**Autism spectrum disorder (ASD)**						
In‑As < 35 μg/g	Reference		Reference		Reference	
In‑As ≥ 35 μg/g	1.12 (0.25, 4.97)	0.882	1.16 (0.25, 5.42)	0.846	1.36 (0.28, 6.49)	0.701
AIC	191.373		194.638		189.827	
**Attention deficit hyperactivity disorder (ADHD)**						
In‑As < 35 μg/g	Reference		Reference		Reference	
In‑As ≥ 35 μg/g	2.91 (1.18, 7.18)	0.020	3.61 (1.38, 9.45)	0.009	3.85 (1.44, 10.29)	0.007
AIC	274.011		276.164		273.655	
**Neurodevelopmental disorders (NDD)**						
In‑As < 35 μg/g	Reference		Reference		Reference	
In‑As ≥ 35 μg/g	2.04 (0.84, 4.93)	0.115	2.64 (1.03, 6.78)	0.043	2.93 (1.11, 7.75)	0.030
AIC	332.023		329.177		320.135	

*Note*: Multiple logistic regression was used; OR, odds ratio; CI, confidence interval; AIC, Akaike information criterion.

*^a^* Urinary In‑As concentration corrected for creatinine μg/g.

*^b^* Model 1, Crude.

*^c^* Model 2, Adjusted for socioeconomic level (mother’s or tutor’s education and the number of people living in the household).

*^d^* Model 3, Adjusted for socioeconomic level (mother’s or tutor’s education and the number of people living in the household), sex, and belonging to indigenous peoples.

## Discussion

The results show a significant association between arsenic exposure and the prevalence of NDDs. Specifically, children with urinary In‑As levels ≥ 35 μg/g creatinine have 2.93 times the likelihood of presenting NDDs and 3.85 times the likelihood of developing ADHD compared to those children with urinary In‑As levels < 35 μg/g.

The median current arsenic concentration was 14.6 μg/L (IQR 9–20.8 μg/L). This level is lower than that previously reported in Arica (median 18 μg/L, IQR 9–28 μg/L) [5], in a study of children evaluated between 2009 and 2015. Some of the differences between the two studies may be related to participant characteristics, such as mean age (10.8 years versus 7.8 years) and the territorial distribution of the sample. In the study by Muñoz et al. [5], 100% of the children were beneficiaries of the Polymetal Law, and their residences were located within a specific area of the city. In contrast, in the present study, only 16% of participants reported being covered by this law, with most coming from various parts of the city. Another plausible explanation for this difference may be the implementation of a new water treatment plant at the beginning of 2013. This new facility contributed to achieving compliance with the Chilean drinking water standards. It could be desirable for future research to consider environmental monitoring data to explore the reasons behind this difference.

The prevalence of NDDs was 12%, like previous studies, where it is reported that 10% of the child population has at least one NDD [12]. Regarding ADHD prevalence (9.1%), it is similar to the 10% described nationally [15] but higher than the 6.4% reported in a previous study in Arica [5]. Since both studies obtained prevalence data through parent self‑reporting, the difference may be due to the sustained increase in ADHD prevalence over time, as in the previous study, the data correspond to records from 2009 to 2014, so there is a 9‑ to 14‑year difference with the current data, reflecting the increase in prevalence described in recent years in other countries, such as the United States [22]. Regarding ASD, the prevalence of 5.3% is higher than described globally (1–2%) [10] and nationally (1.95%) [17]. This difference may be attributed to how the ASD diagnosis data were obtained. In the previous study conducted in Chile, the diagnosis was acquired through clinical observation and the application of the Autism Diagnostic Observation Schedule‑Second Edition (ADOS‑2) as a diagnostic complement. In contrast, the current study employed parent self‑reporting, which may lead to overdiagnosis of the condition. However, it may also indicate that this condition is diagnosed more frequently, so the observed increase is real.

Evidence of the association between arsenic exposure and NDDs in general is limited, with results mainly referring to consequences on IQ [8], cognitive functions [8, 28], and neurocognitive performance [22]. Studies have focused on evaluating associations with ADHD prevalence. In a study conducted on children aged 6 to 7 years in Mexico, children in the third and fourth quartiles of exposure (Q1 = 7.7–35.9; Q2 = 36–55.2; Q3 = 55.3–75.6; Q4 = 75.7–215.9 μg/L) had a twofold higher risk of receiving a score of 6.5 or higher on the ADHD Index [29], meaning that in the Conners’ test, they obtained a high (64–69 points) or very high score (more than 70 points) according to parents and teachers, making it clinically significant for an ADHD diagnosis [30].

In a previous study conducted in Arica, a significant association was observed in the fifth quintile (OR = 2.02, 95% CI 1.12, 3.61) (urinary arsenic range = 31.1–156.0 μg/L) [5]. This result is consistent with what we observed in our sample, where children come across the city, and those with urinary inorganic arsenic levels ≥ 35 μg/g creatinine had 3.85 times the likelihood of developing ADHD (95% CI 1.44, 10.29).

This study has limitations that may have influenced the results. Firstly, it analyzes secondary data; therefore, it was not collected to answer our objective. This may imply the absence of variables of interest, such as clinical data and formal neurodevelopmental diagnoses, as well as other environmental contaminants like lead or cadmium. Similarly, sociodemographic variables, such as household income, would have helped better characterize vulnerability groups. Although the study initially used stratified probabilistic sampling by city sectors based on the 2013–2016 cohort, logistical challenges led to the inclusion of all mothers from that cohort who agreed to participate until the target sample size was reached. Despite this, the spatial distribution of the participants’ residences was adequately represented [26]. Finally, due to the cross‑sectional design, temporality cannot be guaranteed, limiting causal inference. However, despite the described limitations, the results are consistent with previous findings [5, 9].

Among the strengths, using available research technology facilitated data collection and reduced information bias. REDCap significantly reduced missing responses, allowing for nearly complete observations. A second strength is the direct measurement of inorganic arsenic in urine samples, along with its adjustment for creatinine. This allowed for the control of dilution variability, providing a more valid measure of arsenic exposure. Another aspect to highlight is the geographic diversity of the participants’ residences, as children were recruited from various areas across the city. This contributes to the external validity of the findings. Finally, the sample size of 450 children was adequate to achieve a statistical power greater than 80%, reducing the probability of type II error, that is, failing to detect an effect when one truly exists.

The observed association between arsenic exposure and NDD prevalence, where children with urinary inorganic arsenic levels ≥ 35 μg/g creatinine had 2.93 times the likelihood of presenting an NDD (95% CI: 1.11–7.75), represents a relevant finding that contributes to national evidence on arsenic exposure and its consequences on child health. NDDs and their classifications are constantly evolving, with ongoing updates and new associations being identified. Therefore, it is necessary to continue this line of research and address the identified limitations. While the current findings are valuable, they should be interpreted with caution due to the study’s inherent limitations. Future research would benefit from primary data collection, which would enable clinically validated diagnoses, allow the assessment of additional environmental exposures and genetic susceptibility factors, and support the implementation of prospective designs capable of establishing temporality. These methodological enhancements would improve understanding of the mechanisms linking arsenic exposure to neurodevelopmental outcomes.

## Conclusion

An association between arsenic exposure and the prevalence of NDDs was observed in the studied population. A better understanding of this relationship could provide evidence on the effects of arsenic exposure on the health of children exposed to environmental contaminants in Chile, serving as a foundation for informed decision‑making in the future. This knowledge could also support the early identification of children at risk of developing cognitive difficulties, taking advantage of the window of brain plasticity [31], generating effective interactions [32], promoting significant learning opportunities [31], taking early actions [33], and proposing measures to reduce developmental inequalities and minimize the long‑term effects of NDDs throughout life [32].

## Data Availability

Data are not publicly available due to ethical restrictions.
